# Weighted cue integration for straight-line orientation

**DOI:** 10.1016/j.isci.2022.105207

**Published:** 2022-09-30

**Authors:** Shahrzad Shaverdian, Elin Dirlik, Robert Mitchell, Claudia Tocco, Barbara Webb, Marie Dacke

**Affiliations:** 1 Lund Vision Group, Department of Biology, Lund University, 223 62 Lund, Sweden; 2 School of Informatics, The University of Edinburgh, Edinburgh EH8 9AB, UK

**Keywords:** Biological sciences, Zoology, Ethology

## Abstract

Animals commonly integrate multiple sources of information to guide their behavior. Among insects, previous studies have suggested that the relative reliability of cues affects their weighting in behavior, but have not systematically explored how well alternative integration strategies can account for the observed directional choices. Here, we characterize the directional reliability of an ersatz sun at different elevations and wind at different speeds as guiding cues for a species of ball-rolling dung beetle. The relative reliability is then shown to determine which cue dominates when the cues are put in conflict. We further show through modeling that the results are best explained by continuous integration of the cues as a vector-sum (rather than switching between them) but with non-optimal weighting and small individual biases. The neural circuitry in the insect central complex appears to provide an ideal substrate for this type of vector-sum-based integration mechanism.

## Highlights

• The weight of a sun cue for orientation decreases with increasing elevation
• The weight of wind as an orientation cue increases with increasing speed
• Behaviorally, dung beetles integrate cues using a vector summation strategy
• The insect brain is well suited to encode vector summation

## Graphical abstract

**Figure:**
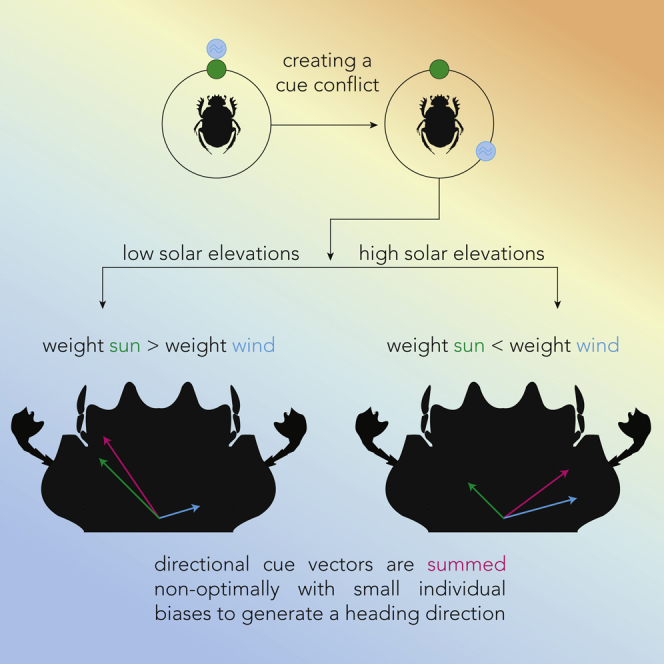

## Introduction

Cue integration is the process whereby an agent combines multiple sensory estimates of the world to perform a single action (Ernst and Bülthoff, 2004). Most explorations of cue integration stem from human psychophysical experiments (Ernst and Banks, 2002; Alais and Burr, 2004), but many examples of cue integration have been found throughout the animal kingdom from monkeys (Fetsch et al., 2012), to rodents (Knight et al., 2014), to insects (Legge et al., 2014; Wystrach et al., 2015; Hoinville and Wehner, 2018; Dreyer et al., 2018; Dacke et al., 2014, 2019; Müller and Wehner, 2007; Collett, 2012; Khaldy et al., 2021; Wehner et al., 2016).

While there is an abundance of evidence suggesting that insects integrate multiple cues when performing navigation behaviors, few propose concrete models which describe the integration process. Compass cue integration presents a direct case study for directional cue integration, and for this, the ball-rolling dung beetle *Kheper lamarcki* (MacLeay, 1821) provides an ideal model organism. Upon finding a dung pat, these beetles break off a piece of dung, shape it into a ball, climb on top of it, and rotate about their own vertical axis. During this “orientation dance”, a snapshot of all available cues is taken and then used to support a directed and efficient escape from the competition at the dung pile (Baird et al., 2012; Byrne et al., 2003; el Jundi et al., 2016). The natural environment provides a plethora of cues that are used by the beetles to sustain this straight-line orientation. Known orientation cues include the position of the sun (Byrne et al., 2003), moon (Dacke et al., 2004), spectral gradients (Jundi et al., 2015), the intensity pattern of the milky way (Dacke et al., 2013; Foster et al., 2017), and wind direction (Dacke et al., 2019). Previous studies have also shown that the beetles will interpret an artificial green light spot as an ersatz sun and will use it to orient with equal accuracy as under natural conditions (El Jundi et al., 2015).

Dacke et al. (2019) previously demonstrated that the influence of a wind cue is dependent on the elevation of the accompanying sun cue. Sun cues above an elevation threshold were seemingly ignored in the presence of a wind cue. This appears to be consistent with the notion that cues are integrated with weights determined by their reliability; cues which are subject to greater perceptual noise (e.g. the azimuth of the sun at a high elevation) are less reliable. Indeed, statistically optimal cue integration models, which seek to maximize the reliability of the combined cue, predict cue weights which are directly proportional to their respective reliabilities (Ernst and Bülthoff, 2004; Murray and Morgenstern, 2010).

Here, we extend this line of work by providing an in-depth exploration of multimodal (ersatz sun and wind) cue integration in the dung beetle compass with an indoor setup which completely isolates the cues under study. Our approach combines behavioral data with computational modeling to describe the cue integration method employed by the South African ball-rolling dung beetle *K. lamarcki*. Our descriptive modeling points to a vector-sum-based integration mechanism, for which the neural circuitry in the insect central complex appears to provide an ideal substrate.

## Results and discussion

### Reliability of ersatz sun and wind cues

**Figure 1:**
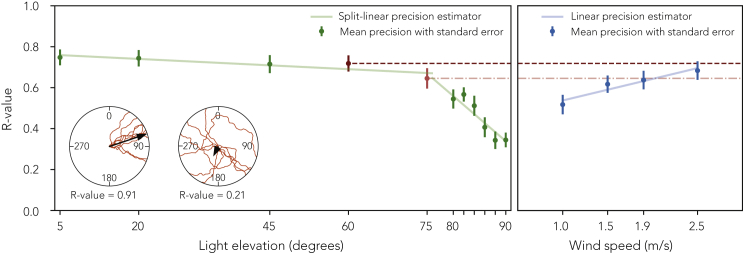
Mean menotactic orientation precision at each elevation and wind speed. Error bars indicate standard error of the mean. The mean precision at $60^{\circ }$ and $75^{\circ }$ elevation is highlighted to allow comparison to the wind (dashed lines). The mean precision at $60^{\circ }$ is close to that at 2.5 m/s wind speed, and similarly the mean precision at $75^{\circ }$ is less than that at 2.5 m/s wind speed but much greater than would be expected at 1.25 m/s. This relationship matches our cue conflict results. The line fits are those used in Equation 2, excluding the additive constants (which are only required for the κ estimation stage, see STAR Methods, Simulated cue representation). Circular insets illustrate ten paths traveled by a highly directed (left, R-value = 0.91) and a weakly directed (right, R-value = 0.21) beetle.

Dung beetle compass orientation is characterized by menotaxis with respect to a given cue-oriented behavior but without a specific directional preference. As a measure of cue reliability, we analyzed beetles’ orientation precision at solar elevations and wind speeds where menotactic behavior was observed with respect to the isolated cue. Wind cues are provided by custom-built wind generators and a solar cue is provided by a green LED (ersatz sun; see STAR Methods, Experimental setup). Cue reliability (variability from the perspective of the animal) is not something we can analyze directly. We therefore use orientation precision as a proxy (see STAR Methods, Simulated cue representation). It is important to note that precision changes could be brought about by changes in a cue’s usefulness, i.e. how good of an orientation cue it actually is from the perspective of the animal, as opposed to its variability. Orientation precision was calculated from ten exit bearings for each beetle and reported as mean vector lengths; the closer to 1 the more clustered the exits, where more clustered exits are interpreted as higher precision (*R*-values; see STAR Methods, Quantification and statistical analysis and Figure 1). To show menotactic behavior statistically, we use Rayleigh tests, where a significant result indicates a directed population (i.e. a preferred direction for all beetles with respect to the cue) and a uniform distribution indicates menotaxis (i.e. arbitrary directions selected by each beetle). These Rayleigh tests were performed on the mean directions taken by beetles where each mean was calculated from the aforementioned ten exit bearings.

### Orientation to the ersatz sun at different elevations

The bearings traveled across the population of beetles at solar elevations of 5°, 20°, 45°, 60°, 80°, 82°, 84°, 86°, and 88° did not differ from a uniform distribution (p= 0.201 (n=18), 0.756 (n=17), 0.534 (n=16), 0.477 (n=17), 0.772 (n=10), 0.237 (n=15), 0.709 (n=9), 0.184 (n=6), and 0.167 (n=4), respectively, Rayleigh tests, see Table S1). Similar results have previously been found in dung beetles orienting under natural conditions (Baird et al., 2010). In contrast, at $75^{\circ }$, the bearings traveled by the beetles were significantly different from uniformity (p=0.02, Rayleigh test, n=13); but as this bearing preference was not present at either lower or higher solar elevations, we decided to treat it as an outlier and proceeded to perform orientation precision analysis for all solar elevations.

The beetles’ ability to maintain their bearings remained stable until an elevation of 75°, beyond which it decreased rapidly with increasing elevation (5° = 0.78 [0.71, 0.84], 20° = 0.78 [0.69, 0.86], 45° = 0.74 [0.64, 0.84], 60° = 0.70 [0.61, 0.88], 75° = 0.69 [0.48, 0.80], 80° = 0.55 [0.40, 0.70], 82° = 0.61 [0.40, 0.66], 84° = 0.50 [0.34, 0.67], 86° = 0.41 [0.27, 0.60], 88° = 0.33 [0.19, 0.48], and 90° = 0.37 [0.22, 0.49], median *R*-values [IQR], n=20, see Figure 1). These results suggest that higher solar elevations provide a less reliable cue, which can be attributed to the decrease in directional information given by a visual cue as it approaches zenith. It should be noted that direct comparisons between the indoor setup and outdoor conditions may not be applicable as the light intensity is lower in the artificial setup. Despite this, a similar decrease in orientation precision at high solar elevations can also be observed outdoors for the same species of dung beetles (Dacke et al., 2014), as well as for other animals (for example, equatorial sandhoppers and desert ants (Ugolini, 2001, 2002; Müller and Wehner, 2007)).

### Orientation to the wind at different speeds

Next, we analyzed the population bearing preference in relationship to the wind cue at different wind speeds. At 0.5, 0.8, 3.0, and 4.0 m/s,the mean bearings differed significantly from a uniform distribution (p<0.05, Rayleigh tests, n= 10, 9, 13, and 14 for wind speeds 0.5, 0.8, 3.0, and 4.0, respectively, see Table S1), suggesting anemotaxis. At 1.0, 1.5, 1.9, and 2.5 m/s, the mean bearings did not differ significantly from a uniform distribution (p= 0.108 (n=7), 0.611 (n=14), 0.734 (n=14), and 0.865 (n=14), respectively, Rayleigh tests, see Table S1), suggesting menotaxis.

Under the conditions where menotaxis was observed, we found that as the speed increased, so did the beetles’ ability to maintain their bearings (1.0 = 0.47 [0.36, 0.72], 1.5 = 0.61 [0.45, 0.80], 1.9 = 0.63 [0.54, 0.81], and 2.5 = 0.68 [0.51, 0.84], median *R*-values [IQR], n=20, see Figure 1). Taken together, the results suggest that higher wind speeds provide a more reliable cue for menotaxis (changing to anemotaxis beyond 3.0 m/s). Previous studies have demonstrated that the antennae of dung beetles are wind sensitive (Linsenmair, 1972), and Okubo et al. (2020) have shown that with increasing wind speed, the antennae of fruit flies are subject to larger displacements. Our results are therefore in line with previous research as one would expect greater deflections of the dung beetle antennae to provide a clearer perception of wind direction. Note the similarity in reliability between a wind cue of 2.5 m/s and an ersatz sun at a 60° elevation (see Figure 1).

### Cue conflict between an ersatz sun and wind

**Figure 2:**
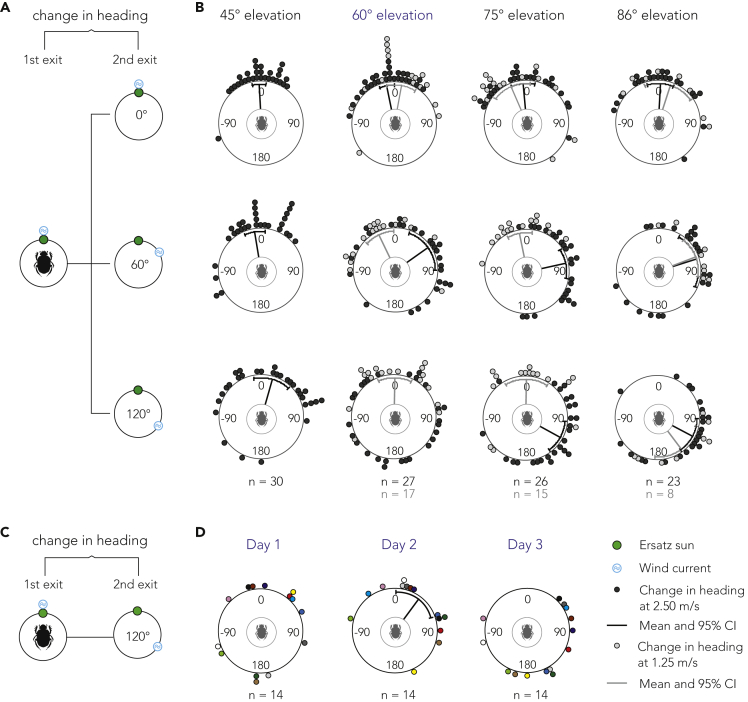
Behavioral results from a cue conflict experiment (A) Schematic procedure of the cue conflict experiment. Change in heading was calculated for individual beetles between two consecutive exits; initial condition (1st exit where the initial bearing is established) to conflict condition (2nd exit where the wind had changed direction by 0°, 60°, or 120° relative to the ersatz sun). (B) The changes in headings at wind speed 2.5 m/s are illustrated as black circles and at 1.25 m/s as gray circles. Lines extending from the centers indicate mean vectors, black lines for 2.5 m/s and gray lines for 1.25 m/s, and end in a 95% confidence interval of the spread. (C) Schematic procedure of the experiment where a 2.5 m/s wind cue was subjected to a 120° azimuthal shift in the presence of a sun cue at 60° elevation. (D) The changes in headings at three different days. Each colored data point illustrates the change in heading of the same individual across days.

The effect of reliability on the integration and weighting of a visual sun cue and a mechanosensory wind cue was studied in a cue conflict experiment. The reliability was manipulated by changing the elevation of the ersatz sun or the speed of the wind current and the conflict was introduced by shifting the azimuthal direction of the wind (see STAR Methods, Behavioural experiments - Cue conflict between an ersatz sun and wind). Changes in heading direction were calculated using the angular difference between two consecutive exits (see Figure 2). All beetles included in the analysis were able to recover their initial bearing following each cue conflict run (see STAR Methods, Quantification and statistical analysis - Cue conflict between an ersatz sun and wind).

#### Dung beetles perform a weighted integration of a wind cue and sun cue

As can be expected from previous studies outdoors (Dacke et al., 2019), when presented with a wind cue of 2.5 m/s and a simulated solar elevation of $45^{\circ }$, $60^{\circ }$, $75^{\circ }$, or $86^{\circ }$, beetles were able to maintain their bearing between two consecutive exits when the directional information from the two cues remained unchanged (μ±SD: $-3^{\circ }\pm 28^{\circ }$ (n=30), $-11^{\circ }\pm 28^{\circ }$ (n=27), $-5^{\circ }\pm 45^{\circ }$ (n=26), and $3^{\circ }\pm 47^{\circ }$ (n=23); p<0.001, Rayleigh tests, see Figure 2B). However, when the directional information from the sun and wind cues were put in conflict, by altering the wind direction between two consecutive exits, the behavior changed depending on solar elevation. At a solar elevation of 45° and the wind current set to 2.5 m/s, the beetles did not change their direction at either a 60° or a 120° cue conflict (μ±SD: $-9^{\circ }\pm 43^{\circ }$ and $16^{\circ }\pm 68^{\circ }$; p<0.001, Rayleigh tests, n=30, see Figure 2B). This suggests that at this elevation, the ersatz sun has a greater weight compared to the wind cue. In contrast, for solar elevations of $75^{\circ }$ and $86^{\circ }$, the beetles updated their bearing in accordance with the 60° or 120° azimuthal change of the wind current (μ±SD: $78^{\circ }\pm 56^{\circ }$ and $116^{\circ }\pm 60^{\circ }$; p<0.001 at $75^{\circ }$ elevation (n=26), $74^{\circ }\pm 80^{\circ }$ and $123^{\circ }\pm 62^{\circ }$; p=0.025 and p<0.001 at $86^{\circ }$ elevation (n=23), Rayleigh tests, see Figure 2B). At a 60° solar elevation, the beetles again responded to the 60° azimuthal shift of the wind (μ±SD: $55^{\circ }\pm 76^{\circ }$; p<0.01, Rayleigh test, n=27); interestingly, when presented with a conflict of $120^{\circ }$, the changes in bearing did not differ significantly from a uniform distribution (p=0.837, Rayleigh test, n=27). Thus, the beetles did not seem to keep their relative bearing to either the ersatz sun or the wind.

Together, our results suggest that the relative weight between the sun cue and the wind cue is affected by elevation and that the critical elevation (at which the ersatz sun becomes less reliable than a wind cue of 2.5 m/s) lies between 45° and 75°. This result matches previous observations in dung beetles (Dacke et al., 2019) and ants (Müller and Wehner, 2007); the higher the solar elevation, the lower the influence of the cue. From our results, it may appear as if the beetles are simply following the more reliable cue, which has previously been a suggested orientation strategy among dung beetles when presented with sun and wind (Dacke et al., 2019), sun and polarized light (Jundi et al., 2014), or sun and other skylight cues (Dacke et al., 2014). However, considering the uniform distribution found at a solar elevation of 60° together with a 120° cue conflict, the beetles are not always able to follow one cue over the other. This could indicate that at this elevation, the cue reliabilities intersect. Furthermore, the population spread increases with conflict; this effect is consistent across almost all test conditions when the wind speed is set to 2.5 m/s. This suggests that the beetles are not following a simple winner-take-all strategy, as under strict winner-take-all (see STAR Methods, Integration models) the population dispersion should be unaffected by cue conflict. The pseudo winner-take-all behavior and increasing variance that we observe at elevations of 45°, 75°, and 86° could be explained by a circular integration model (Murray and Morgenstern, 2010) with non-optimal weights (see STAR Methods, Integration models), suggesting both cues are contributing to behavior, even when one appears to be followed and the other ignored.

#### Orientation behavior varies when cue weights are similar

Due to the random distribution of beetles’ changes in heading when the 60° elevation sun cue and the 2.5 m/s wind cue were put in conflict by 120°, it appeared that the population failed to orient under this condition. However, an additional experiment that focused on individual precision, in which each beetle was permitted to exit the arena ten times in the presence of the cue conflict conditions, showed that the beetles did not fail to orient. Instead, we found that the beetles oriented along a new random bearing that they then successfully maintained (0.91 [0.86, 0.95], median *R*-value [IQR], n=11). Beetles were also able to maintain their bearings at conflicts of 0° and 60° (0.76 [0.66, 0.85] and 0.89 [0.82, 0.91], respectively, median *R*-value [IQR], n=11).

Furthermore, when the two cues were returned to their original positions, the beetles recovered their initial bearing (see STAR Methods, Quantification and statistical analysis - Cue conflict between an ersatz sun and wind), suggesting that this new random bearing we see is actually an effect of the integration strategy and not a permanent re-set of the bearing. Similarly, Khaldy et al. (2021) showed that the ball-rolling dung beetles *Garreta unicolor* and *Garreta nitens* appeared disoriented when subjected to a conflict produced by simultaneous manipulation of a sun cue and the pattern of polarized light. However, upon returning the cues to their original positions, these animals recovered their initial bearings. Likewise, bogong moths fly in a seemingly disoriented manner when presented with a conflict between the magnetic field and visual landmarks; when the cues are returned to their original positions the moths, too, recover their initial bearings (Dreyer et al., 2018).

To explore these apparent new bearings taken by beetles at a 120° conflict, we tested whether the individual change in bearing was consistent over different days or if it was prone to change. We employed the previously described cue conflict assay, focusing on a $60^{\circ }$ solar elevation with a wind speed of 2.5 m/s and tested individual beetles over three consecutive days. Similar to our previous cue conflict experiments, the beetles were able to maintain their bearings each day when the cues were kept in their original positions (μ±SD: $-1^{\circ }\pm 23^{\circ }$, $9^{\circ }\pm 41^{\circ }$, and $-4^{\circ }\pm 44^{\circ }$ for day one, two, and three, respectively; p<0.001, Rayleigh tests, n=14, see Figure S3). However, upon changing the azimuthal position of the wind by 120°, the individual change in bearing differed across the three days (n=14, see Figure 2D). This shows that the apparent new bearing taken by beetles at a $60^{\circ }$ solar elevation is not consistent over days.

The results from this three day experiment reinforce the idea that a weighted integration is taking place as both cues must be considered to generate the variability we see in the mean vector (see Figure 2D); if this were a winner-take-all (or biased winner-take-all), we would not expect to see any beetles in the lower left-hand quadrant. Furthermore, the inconsistency in population response suggests an additional source of noise in the integration process. A potential explanation for this noise would be individual variation or “preference”. A very small (random) individual bias could cause an increase in spread in the population of responses where cue weights are near equal (see STAR Methods, Integration models; Results and Discussion, Modeling).

#### The weight given to a sun cue and a wind cue is dictated by their relative reliability

In previous experiments, the reliability of the ersatz sun was altered. Here, the wind speed was reduced to 1.25 m/s to study the effect of decreased wind reliability on the relative weighting of the sun and wind cues. We again employed the previously described cue conflict assay at solar elevations $60^{\circ }$, $75^{\circ }$, and $86^{\circ }$.

At solar elevations of $60^{\circ }$ and $75^{\circ }$, the directional changes of the wind current had no effect on the beetles’ orientation behavior, regardless of conflict angle (μ±SD: for conflicts $0^{\circ }$, $60^{\circ }$, and $120^{\circ }$: $12^{\circ }\pm 46^{\circ }$, $-25^{\circ }\pm 46^{\circ }$, and $1^{\circ }\pm 51^{\circ }$ for $60^{\circ }$ elevation (n=17), $-24^{\circ }\pm 55^{\circ }$, $-11^{\circ }\pm 33^{\circ }$, and $1^{\circ }\pm 58^{\circ }$ for $75^{\circ }$ elevation (n=15); p<0.01, Rayleigh tests, see Figure 2). However, at an $86^{\circ }$ solar elevation, the beetles changed their bearings in accordance to the azimuthal shift of the wind (μ±SD: $17^{\circ }\pm 51^{\circ }$, $68^{\circ }\pm 35^{\circ }$, and $143^{\circ }\pm 48^{\circ }$; p=0.023, p<0.01 and p=0.013, with increasing conflict, Rayleigh tests, n=8, see Figure 2). These findings stand in contrast to the behavior observed in the presence of a 2.5 m/s wind current at the same solar elevations (recall, at a wind speed of 2.5m/s and solar elevation of $75^{\circ }$ the beetles attributed a higher relative weight to the wind). These results further reinforce the conclusion that relative reliability dictates the weight given to each individual cue.

#### Behavior indicating a weighted integration strategy

In all, our behavioral results show that the dung beetle compass is dynamic and that relative cue reliability dictates which cue is favored; this occurs as a continuous integration process, rather than a winner-take-all. The cue which is perceived to be less noisy, and thus more reliable, is predominantly used to guide straight-line orientation. This holds true until the relative reliabilities are similar and the beetles initially appear to be unable to utilize the provided cues for orientation. However, when investigating individual precision, we found that beetles were able to maintain their apparent new bearing, as well as recover their initial bearing when the cues were returned to their original positions. Furthermore, individual orientation behavior differed across three days. Together, these results suggest that the observed randomness is an effect of a weighted integration strategy, but the integration may be inconsistent across days. To attempt to characterize the integration strategy, we performed simulations to experiment with different strategies and weight relationships.

### Modeling

**Figure 3:**
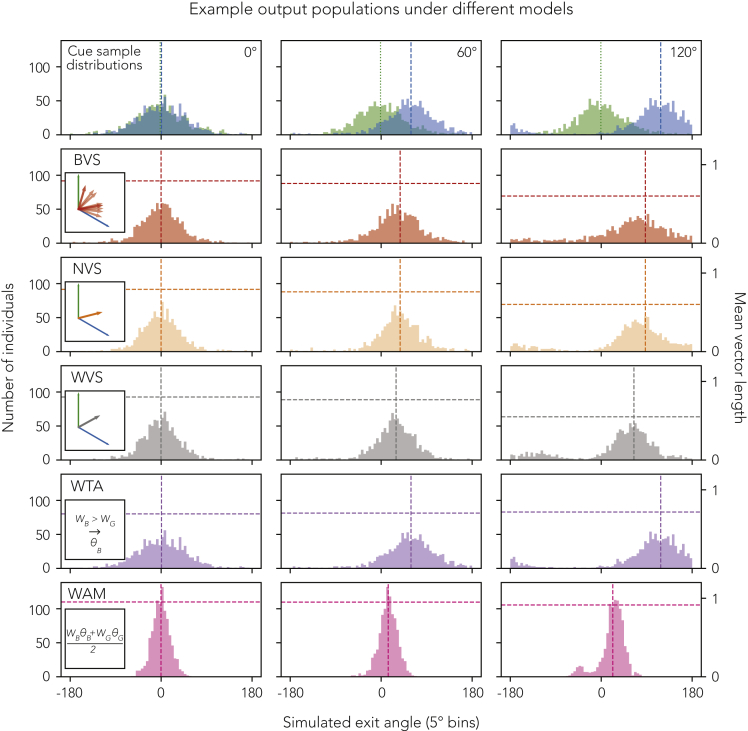
An illustration of the different model outcomes for two sets of input samples Top row: The two sample distributions used as input to the models. Each cue is described by a noise distribution which is sampled to generate behavior. The noise distributions are von Mises with $\kappa_{Blue}=2.05$, $\kappa_{Green}=2$, $\mu_{Green}=0$, and $\mu_{Blue}\in \left\{0^{\circ }\text{,}60^{\circ }\text{,}120^{\circ }\right\}$ (columns). Weights are computed from κs. **BVS** (Biased Vector Sum): Noise is added to the weights (vector magnitudes) which are then passed through an adjustment function. This strategy can generate different outputs for the same inputs due to the added noise. **NVS** (Non-optimal Vector Sum): Weights are adjusted and then the vectors are summed. **WVS** (Weighted Vector Sum): Angular samples are converted to vectors and then summed. **WTA** (Winner-take-all): Weights are compared and the cue with the greatest weight wins complete influence. **WAM** (Weighted Arithmetic Mean): A simple weighted average of the angles. For model definitions, please see STAR Methods, Integration models.

Cue integration studies typically compare winner-take-all (WTA) to “optimal” cue integration, defined as the linear weighted arithmetic mean, WAM (Ernst and Banks, 2002; Ernst and Bülthoff, 2004), or the circular weighted vector sum, WVS (Murray and Morgenstern, 2010). Previous work on dung beetles has assumed WTA (Dacke et al., 2019) but, as already noted, several aspects of our current results do not appear to match what this model would predict. Some studies on ant behavior have been taken to demonstrate WAM (Legge et al., 2014; Sun et al., 2018; Wystrach et al., 2015), but WAM is inappropriate for circular inputs. Instead, cue integration in the circular domain can be represented by a weighted vector sum (STAR Methods, Integration models), for which the optimal weights (which minimize the variance of the combined cue) are given by the concentrations of the von Mises noise distributions which characterize each cue. WVS has been used in a model of ant navigation (Hoinville and Wehner, 2018), and has the interesting property that it can resemble WAM at small conflicts and WTA at large conflicts (see Figure S6). This has inspired us to consider two further alternatives. The first is a “non-optimal weighted” vector sum (NVS) which exaggerates the pseudo-WTA region of the WVS, such that the stronger cue usually dominates the response but both contribute, unlike true WTA. The second is a biased (non-optimal weighted) vector sum (BVS), which simulates small individual biases toward one or the other cue, creating a variety of behavior when the cues are near-equally balanced. Illustrative model outputs for the same input distributions are given in Figure 3. For completeness, we compare all five models (WTA, WAM, WVS, NVS, and BVS, defined explicitly in STAR Methods, Integration models) to the behavioral data to calculate their relative likelihoods. The results are given in Table 1.

**Table 1:** Cross-model comparison results in order of likelihood/fit

Model	Parameters	Log likelihood ratio	AIC ratio	BIC ratio
**BVS**	2	0.00000	1.000000	1.000000
**NVS**	1	−17.21685	1.007461	1.006451
**WVS**	0	−50.45796	1.022295	1.020260
**WTA**	0	−50.73313	1.022421	1.020386
**WAM**	0	−199.68191	1.090951	1.088779

All circular models (WVS, NVS, and BVS) outperform the weighted arithmetic mean (WAM). Non-optimal weighted circular models (NVS and BVS) do better than both the statistically optimal WVS and previously hypothesized WTA. As parameter counts are small, the Akaike information criterion (AIC) and Bayesian information criterion (BIC) do not sufficiently penalize either BVS or NVS to affect the order of the results. Likelihoods are best at 0 and decrease as models become less likely. The AIC and BIC ratios are best at 1 and increase as goodness-of-fit decreases.

Our modeling results indicate that all circular models (WVS, NVS, and BVS) better account for our behavioral data than the classically considered WAM and WTA. The extremely similar outcomes between WTA and WVS were unexpected, but it is likely that these models both account for different portions of the data (i.e. they are equally bad at capturing the full range of behavior). The non-optimal circular model (NVS), which takes advantage of the pseudo-WTA property of a circular integration model (a vector sum, see STAR Methods, Integration models), performs substantially better than either optimal circular integration or a winner-take-all as this model should capture the small influence of the secondary cue. Finally, the inclusion of individual bias in BVS increases the population-level noise where cue weights are near equal, which should capture the behavioral variability at the critical elevation conditions ($60^{\circ }$ elevation, 2.5 m/s wind speed), leading to the best overall fit.

The key take-away from our simulations is that a weighted circular model best accounts for the data. WVS, NVS, and BVS make different behavioral predictions. WVS predicts mostly intermediate courses with the more reliable cue dominating at large conflicts. NVS predicts the more reliable cue will dominate most of the time with intermediate courses where cue weights are similar. Finally, BVS predicts mostly dominance, with some intermediate courses where cue weights are similar, and increased population variance where they are near equal. Despite their differences, these three models use the same basic vector sum (WTA can also be represented as an extreme case of NVS with very large *a*, see STAR Methods, Integration models). They differ only in how their weights are computed.

To tie our results to physiology, the neural circuitry in the insect central complex appears to provide an ideal substrate for performing a vector sum calculation. In the insect brain, head direction is maintained by a ring-attractor circuit (Seelig and Jayaraman, 2015; Kim et al., 2017; Heinze, 2017). This circuit is fed by the ring neurons, which seem to cluster into groups which are sensitive to different orientation cues (Okubo et al., 2020; Hardcastle et al., 2021). The two layers are linked by plastic all-to-all connections—every ring neuron connects to every compass (head direction) neuron (Kim et al., 2019; Fisher et al., 2019)—which should allow the network to learn relationships between different cues, forming a single integrated snapshot for orientation. In beetles, the relationship between the different available cues could be learned during the dung beetle “dance” (Baird et al., 2012), which is thought to be the point at which their orientation snapshot is taken (el Jundi et al., 2016). Similar neural models of angular cue integration have been proposed by Page et al. (2014) and Jeffery et al. (2016) in rodents, and Sun et al. (2018) in insects. Interestingly, Sun et al. (2018) implement WAM within a ring-attractor model and observe a strategy switch (where the output switches from WAM to WTA), which looks strikingly similar to the integration curves shown by Murray and Morgenstern (2010) (Figure S6, Right). Working with rodents, Knight et al. (2014) also note a “switch” between apparently optimal integration behavior and cue selection behavior and finally, Sjolund et al. (2018) note similar results for spatial (distance and direction) cue integration in humans. Not only is a weighted circular model a likely candidate for cue integration in our case and in ants (Hoinville and Wehner, 2018), but the orientation center of the insect brain would seem to be well suited to encode the underlying vector sum. Overall behavior is then governed by the weights used, which need not be consistent across different species.

In summary, our behavioral data point to a weighted integration of wind and solar cues. Subsequent computer modeling suggests that the integration is most likely a form of vector summation, which seems to be well encoded by the insect head direction circuit. Vector summation can produce a variety of different integration outcomes depending on the weights used; a neural circuit which supports vector summation could produce different behavior depending on how an agent computes these weights. Thus, a single core model (vector summation) with different peripheral processing stages (weight-adjustment and/or bias etc.) may explain a wide range of cue integration behaviors across different insect species, despite the highly conserved neuroanatomy.

### Limitations of the study

To fully isolate the orientation cues in question (sun and wind cues), we performed our experiments in an indoor setup which allowed us to control all possible cue parameters. Consequently, the indoor setup is limited in its representation of the real world. One major constraint of our behavioral setup is motion parallax, which becomes more severe at higher solar elevations.

The modeling is based on beetle exit angles which do not fully characterize the strategy in use by the beetles (especially with the aforementioned motion parallax). Modeling based on full tracks for each individual would be more informative but these data are not practically available. This means that applications of the specific model instances presented are limited.

## STAR★Methods

### Key resources table

**Table:** 

REAGENT or RESOURCE	SOURCE	IDENTIFIER
**Deposited data**		

Exit angles/behavioral data	This study	Zenodo: 10.5281/zenodo.5724225
Model simulated data	This study	Zenodo: 10.5281/zenodo.5724225

**Experimental models: Organisms/strains**		

Diurnal *Kheper lamarcki*	Wild caught	MacLeay, 1821

**Software and algorithms**		

Python	Open source	Version 3.8.10
Oriana	Kovach Computing services, UK	Version 3.21

**Other**		

Handycam	Sony, Japan	FDR-AX53

### Resource availability

#### Lead contact

Further questions should be directed to and will be answered by the lead contact, Elin Dirlik (elin.dirlik@biol.lu.se).

#### Materials availability

This study did not generate new unique reagents.

### Experimental model and subject details

#### Ethical statement

All applicable international, national and institutional guidelines for the care and use of animals were followed. Animal care was in accordance with the EU Directive 2010/63/EU and the South African National Standard for The Care and Use of Animals for Scientific Purposes.

#### Animal collection and experimental sites

Ball rolling dung beetles of the species *Kheper lamarcki* were collected using dung baited pit-fall traps at Stonehenge game farm, South Africa (26.39° S, 24.32° E) during November 2019, as well as February and November 2020. Behavioural experiments were conducted indoors at Bergsig Eco Estate game farm near Bela Bela and at University of the Witwatersrand, South Africa, as well as at Lund University, Sweden. Beetles were kept in plastic containers filled with sand and provided with fresh dung 2–4 times per week.

### Method details

#### Experimental setup

The setup consisted of an artificial sky constructed of two metal arches crossed over to create a hemisphere (1.5 *m* radius). Each arch was lined with 141 LEDs (520 nm, DotStar; Adafruit Industries, New York, USA) approximately 1.3° apart. A single LED served as an ersatz sun with an intensity of $2\times 10^{11}photons/cm^{2}/sec$ as measured from the centre of the setup at a height of about 7 cm (corresponding to the height of a beetle on top of its dung ball) using a spectrometer (QE65000; Ocean Optics). Serving as wind cues, four wind generators were positioned on the floor 1.3 *m* from the centre of the setup. The first wind generator was aligned with one of the LED-lined arches and the remaining three were placed at an angle of 60°, 120° and 180° relative to the first. Each wind generator was constructed from three fans (PFR0912XHEE, 4.50*A*; Delta Electronics Inc., Taipei City, Taiwan) separated by 0.25 *m* and was powered by a Mean Well RSP-320-12, 26.7*A* power supply. Measures of wind speeds were obtained by the use of a hot wire anemometer (HHF-SD1; Omega) placed 7 cm above the centre of the arena (see Figure S4). A sand-painted circular arena (0.3 *m* radius) was placed in the centre of the setup, with the arena perimeter labelled from 0 to 355° in 5° increments and with 0° aligned with magnetic north. To control solar elevation and wind speed, custom-built software was used with an Arduino Uno (experiments conducted in South Africa), or a Raspberry Pi 4 Model B (experiments conducted in Sweden). All experiments were filmed from above using a Sony camera (FDRAX53 Handycam) or a Raspberry Pi camera (Camera Module 2 NoIR), supported by infrared illumination (B07DDJ1YDB, 1A; eecoo, Shenzhen, China). To eliminate unwanted cues, the setup was placed inside a tent made out of blackout cloth (see Figure S1).

#### Behavioural experiments

Throughout each experimental day beetles were temporarily kept in shallow bins containing fresh dung and given time to construct dung balls. During behavioural experiments, each beetle was placed alongside its dung ball in the centre of the circular arena (semi-randomly in one of four cardinal directions). Following its characteristic orientation dance, the beetle was allowed to roll to the perimeter where its exit bearing was recorded. The beetle and its ball were then placed back into the centre of the arena and the procedure was repeated a number of times that depended on the experimental question (see below). In total, each beetle took between 5 and 15 min to complete an experimental series, after which it was put away for the day. The same beetle was never tested more than once for each experiment and if it performed another experimental series this was always carried out on a different day.

##### Reliability of sun and wind cues

We used orientation precision of ball rolling beetles as a proxy for reliability under different cue conditions with the assumption that more reliable cues would lead to greater precision and vice versa. Orientation precision under an ersatz sun was tested at elevations of 5°, 20°, 45°, 60°, 75°, 80°, 82°, 84°, 86°, 88°, or 90°. For every elevation, 20 beetles were tested. Each individual was marked to ensure that it was only used once per elevation. Each beetle was placed in the center of the arena and allowed to exit from it five times. Following this, the azimuth of the ersatz sun was shifted by 180° and the beetle exited the arena an additional five times.

The same procedure was used to test the beetles’ ability to perform straight-line orientation at wind speeds of 0.5, 0.8, 1.0, 1.5, 1.9, 2.5, 3.0 and 4.0 m/s, with the direction of the wind current shifted by 180° after five exits. For every wind speed 20 beetles were tested and each individual was marked to ensure that it was only tested once per wind speed. To sustain the beetles’ motivation in the presence of wind, this experiment was performed with an ersatz sun positioned in zenith.

##### Cue conflict between an ersatz sun and wind

###### Cue conflict experiments

Based on the results gathered from the reliability experiments described above, a cue conflict experiment was conducted using an ersatz sun at elevations of 45°, 60°, 75°, and 86° in the presence of a wind current of 2.5 m/s. In a separate cue conflict assay, solar elevations of 60°, 75° and 86° were presented together with a wind speed of 1.25 m/s. All conflicts were achieved by shifting the azimuthal direction of the wind current while keeping the ersatz sun stationary.

Each beetle exited the arena a total of eight times: three times with the directional information from the ersatz sun and wind current in congruence, once with a conflict of $60^{\circ }$ (or $120^{\circ }$), once with the cues in their original position (congruent), once with a conflict of $120^{\circ }$ (or $60^{\circ }$, respectively), and finally two exits with the cues returned to their original positions (congruent). The purpose of the repeated congruent exits was to ensure that the beetles strived to adhere to the same bearing throughout its experimental series (see STAR Methods, Quantification and statistical analysis - Cue conflict between an ersatz sun and wind). The order in which the conflicts were presented was pseudo-randomised. Thus, each beetle performed both test conditions where the azimuthal directional information of the two cues were put in conflict by 60° or 120° between two consecutive rolls, as well as a control condition where the directional information remained unchanged (0° conflict).

###### Further cue conflicts at 60° elevation

The same cue conflict assay (congruent (×3) - 60°/120° conflict - congruent - 120°/60° conflict - congruent (×2)) was replicated in another experimental series. These spanned over three days with the ersatz sun at an elevation of 60° and the wind speed set to 2.5 m/s. Each day, data was collected from the same population of individually marked beetles.

###### Individual precision

Individual precision was studied at an elevation of 60° and a wind speed of 2.5 m/s. In this experimental setting, the beetles exited the arena a total of 36 times: ten times with an ersatz sun and a wind current in congruence, ten times with a conflict of $60^{\circ }$ (or $120^{\circ }$), three times with the cues in their original positions, ten times with a conflict of $120^{\circ }$ (or $60^{\circ }$, respectively), and finally three times with the cues returned to their original positions.

#### Simulation overview

The software performs a simplified simulation of the cue conflict paradigm above. We are interested in the change in the value of an integration of two angular inputs with von Mises noise. We define a von Mises distribution for each cue (using the precision data described in STAR Methods, Behavioural experiments - Reliability of sun and wind cues) and an angle is sampled from each. We then compute the integration of these angles when the cues are aligned (their distributions have the same mean), and when the cues are in conflict (the distributions have different means). The difference between the two integrations is the change in the integration, which can be interpreted as a change in bearing. We compared five different cue integration models and assessed their ability to produce our behavioral data by comparing how likely the data would be under any candidate model (see STAR Methods, Evaluation process).

#### Simulated cue representation

In order to capture sensory noise in a circular context, cues are treated as independent von Mises random variables (Murray and Morgenstern, 2010). The von Mises probability density function is given by:

(Equation 1) $$f_{VM}\left(x;\mu \text{,}\kappa \right)=\frac{e^{\kappa cos\left(x-\mu \right)}}{2\pi I_{0}\left(\kappa \right)}$$

where μ is the mean angle of the distribution, κ is the concentration (equivalent to $\sigma^{-2}$ for the normal distribution, often called “reliability” (Ernst and Bülthoff, 2004; Murray and Morgenstern, 2010)), and $I_{0}\left(a\right)$ is the modified Bessel function of the first kind of order zero (Batschelet, 1981; Murray and Morgenstern, 2010). This is analogous to using normal distributions to simulate Gaussian noise when working with linear data (e.g. time taken by an animal to exit an arena), rather than angular data (e.g. angle at which the animal exits the arena). To sample from these distributions we need to estimate parameters $\mu_{Wind}$, $\kappa_{Wind}$, $\mu_{Light}$, $\kappa_{Light}$, such that the distributions $f_{Light}\left(x;\mu_{Light}\text{,}\kappa_{Light}\right)$ and $f_{Wind}\left(x;\mu_{Wind}\text{,}\kappa_{Wind}\right)$ are those which can produce simulations which match the observed behavior under light (an ersatz sun) or wind respectively.

The estimates for the means are the input azimuths of each cue; it is reasonable to assume that the average perceived cue position is the true cue position. The concentration parameter estimates can be approximated from the mean vector length of a random sample from a parent distribution (Mardia and Jupp, 2009). The best available proxy for such a random sample is the data collected to examine the reliability of sun and wind cues (see Results and Discussion, Reliability of ersatz sun and wind cues). We tried to model the reliability data using linear and split-linear fits respectively (performed using SciPy curve-fitting utilities (Virtanen et al., 2020)); these fits can be seen in Figure 1. However, if we try to approximate the κ-values from these directly the resultant populations are less precise than they should be; to fix this, we included small additive constants which augment the mean vector lengths, improving the final κ approximation with respect to the observed data. The final estimators are:

(Equation 2) $$R_{Wind}=\left(0.11s+0.43\right)+c_{Wind}$$

(Equation 3) $$R_{Light}=\left\{ \begin{array}{ll} \left(-0.07\varphi +0.80\right)+c_{Light}, & \text{if }\varphi \leq 75^{\circ },\\ \left(-1.26\varphi +2.31\right)+c_{Light} & \text{otherwise}. \end{array}\right.$$

where *s* is wind speed, φ is light elevation with additive constants $c_{Light}=0.135$, and $c_{Wind}=0.133$. $c_{Light}$ and $c_{Wind}$ were tuned by hand.

We can now estimate an augmented mean vector length *R* for any sensible light elevation or wind speed which we can use to approximate $\hat{\kappa }$, from Mardia and Jupp (2009) (pg. 85, 86):

(Equation 4) $$\hat{\kappa }\approx \left\{ \begin{array}{cc} 2R+R^{3}+\frac{5}{6}R^{5}, & \text{if }R<0.53\\ \frac{1}{2\left(1-R\right)-{\left(1-R\right)}^{2}-{\left(1-R\right)}^{3}}, & \text{if }R\geq 0.85\\ -0.4+1.39R+\frac{0.43}{\left(1-R\right)}, & \text{otherwise} \end{array}\right.$$

The quality of this approximation can be seen in Figure S7; the approximation is slightly faster to compute, saving some time when running larger simulations. We can test the κ-values by simulating the precision experiments used to estimate them. Including the additive corrections, this method allows us to simulate beetle populations which approximately match real beetles under single-cue conditions.

#### Integration models

With the above cue representation, we compared five different simple models to evaluate how likely they are to have produced the experimental data. Each integration is computed twice per simulated individual; once for the initial condition and once for the conflict condition.

##### Winner-take-all (WTA)

Under winner-take-all, we compute weights for each cue and the integration is simply the cue azimuth of the cue with the greatest weight. Weights and integration are given by:

(Equation 5) $$W_{Wind}\leftarrow \kappa_{Wind}$$

(Equation 6) $$W_{Light}\leftarrow \kappa_{Light}$$

(Equation 7) $$I_{WTA}=\left\{ \begin{array}{c} \theta_{Wind}\text{ if }W_{Wind}>W_{Light},\\ \theta_{Light}\text{ otherwise}. \end{array}\right.$$

Note we do not check the case where cues have equal weights because this never occurs. In such an instance you could break the tie randomly.

##### Weighted arithmetic mean (WAM)

WAM is the standard (statistically optimal) weighted average model which arises throughout cue integration literature (Ernst and Banks, 2002; Ernst and Bülthoff, 2004; Knill and Pouget, 2004). A weighted arithmetic mean is not appropriate for angular or otherwise cyclic inputs (Batschelet, 1981; Murray and Morgenstern, 2010); a standard example in circular statistics is to consider the average of $0^{\circ }$ and $360^{\circ }$. If we assume equal weights, then Equation 10 will give $180^{\circ }$ where we would expect $0^{\circ }$. However, this method has previously been used in the context of directional cue integration in ants (Sun et al., 2018; Wystrach et al., 2015), humans (Alais and Burr, 2004), and monkeys (Fetsch et al., 2012). Furthermore, direction and distance can often be mixed and discussed generally as ‘spatial’ cues (Cheng et al., 2007; Nardini et al., 2008; Sjolund et al., 2018) which can lead to difficulty when interpreting integration across two different domains. Thus, due to its widespread application, we included WAM in our comparison. The weights and integration are given by:

(Equation 8) $$W_{Wind}\leftarrow \kappa_{Wind}/\left(\kappa_{Wind}+\kappa_{Light}\right)$$

(Equation 9) $$W_{Light}\leftarrow \kappa_{Light}/\left(\kappa_{Wind}+\kappa_{Light}\right)$$

(Equation 10) $$I_{\text{WAM}}=W_{Wind}\theta_{Wind}+W_{Light}\theta_{Light}$$

##### Weighted vector sum (WVS)

This method (due to Murray and Morgenstern (2010)) is derived from a Bayesian integration of angular cues with von Mises noise. Its function is best understood by considering a vector sum heuristic; if we interpret each cue as a polar vector, say $\bar{l}=\left(\theta_{Light}\text{,}W_{Light}\right)$ and $\bar{w}=\left(\theta_{Wind}\text{,}W_{Wind}\right)$, then Equation 13 gives the angular component of $\bar{l}+\bar{w}$ (Murray and Morgenstern, 2010). This method has been used previously to model the integration of Path Integration and Landmark Guidance cues in ants (Hoinville and Wehner, 2018). The weights are:

(Equation 11) $$W_{Wind}\leftarrow \kappa_{Wind}/\left(\kappa_{Wind}+\kappa_{Light}\right)$$

(Equation 12) $$W_{Light}\leftarrow \kappa_{Light}/\left(\kappa_{Wind}+\kappa_{Light}\right)$$

The final integration is:

(Equation 13) $$I_{\text{WVS}}=\theta_{Wind}+\text{atan}2\left(\text{sin}\left(\theta_{Light}-\theta_{Wind}\right)\text{,}\left(W_{Wind}/W_{Light}\right)+\text{cos}\left(\theta_{Light}-\theta_{Wind}\right)\right)$$

So long as $W_{Wind}/W_{Light}=\kappa_{Wind}/\kappa_{Light}$, this integration is considered optimal (Murray and Morgenstern, 2010; Hoinville and Wehner, 2018). The following methods are variations on this vector sum which differ only in how the weights are computed (i.e. the magnitude components of the vector arguments).

##### Non-optimal weighted sum (NVS)

Here we compute the normalised weights as in WAM and WVS, then pass them through a sigmoid adjustment function *g*. The adjustment function has the effect of minimising the area in weight-space where both cues have a significant impact on the integration (see Figure S5). Thus, both cues are still considered but it is quite easy for one to dominate the integration resulting in pseudo-WTA behavior. This is distinguished from a true WTA by the fact that increasing cue conflict could still have an effect on overall population spread (as both cues are generally considered, even if one has very little weight).

(Equation 14) $$W_{Wind}\leftarrow g\left(\kappa_{Wind}/\left(\kappa_{Wind}+\kappa_{Light}\right);a\right)$$

(Equation 15) $$W_{Light}\leftarrow g\left(\kappa_{Light}/\left(\kappa_{Wind}+\kappa_{Light}\right);a\right)$$

where:

(Equation 16) $$g\left(x;a\right)=\frac{1}{1+e^{-a\left(x-0.5\right)}}$$

with a=53 determined as most likely (see STAR Methods, Evaluation process; Figure S8). The final integration is given by Equation 13.

##### Biased non-optimal weighted sum (BVS)

Our final model introduces small individual biases which give each individual a random preference for the wind or the ersatz sun. These biases are drawn from a very narrow Gaussian distribution ($\sigma_{Bias}^{2}=0.000303$) with μ=0. As such, we expect that most individuals have no bias. In biological terms, this could be thought of as a weak preference based on prior experience; such a preference would only become apparent where cues are very close in weight. The weights are now computed as:

(Equation 17) $$W_{Wind}\leftarrow g\left(\left(\kappa_{Wind}/\left(\kappa_{Wind}+\kappa_{Light}\right)-b\right);a\right)$$

(Equation 18) $$W_{Light}\leftarrow g\left(\left(\kappa_{Light}/\left(\kappa_{Wind}+\kappa_{Light}\right)+b\right);a\right)$$

where $b∼N\left(0,\sigma_{Bias}=0.017\ldots \right)$ is the bias for this individual. Note that the same bias is used on both the initial and conflict steps. Again, the final integration is given by Equation 13.

#### Evaluation process

To evaluate the different models, we used each to generate large simulated populations ($n_{sim}=1,000,000$) under each set of conflict conditions (for each model, the same von Mises random samples were used to minimise the effect of random sampling when comparing models). These populations can be interpreted as probability mass functions (grouped into $5^{\circ }$ bins and then normalised) and used to assess how likely each model is to have produced the experimental data above. We cannot simply fit von Mises distributions to our data as: (1) the integration of two von Mises distributions does not necessarily produce a von Mises distribution (our models do not necessarily produce von Mises distributions) (Murray and Morgenstern, 2010), and (2) three of our test conditions did not produce significantly oriented populations (Figure 2B, 60° elevation/120° conflict, and Figure 2D Day 1 and Day 3) meaning we cannot assume they are von Mises.

Formally, given data for a set of conditions *c*, and a model *M* we want to know:

(Equation 19) P(M|c)∝P(c|M)P(M)

(Equation 20) P(M|c)∝P(c|M)

as P(M) is assumed to be uniform. We have 21 different conditions, each of which is conditionally independent, thus:

(Equation 21) $$P\left(c|M\right)=\overset{21}{\underset{j=1}{\prod }}P\left(c_{j}|M\right)$$

(Equation 22) $$\text{ln}\left(P\left(c|M\right)\right)=\text{ln}\left(\overset{21}{\underset{j=1}{\prod }}P\left(c_{j}|M\right)\right)$$

(Equation 23) $$=\overset{21}{\underset{j=1}{\sum }}\text{ln}\left(P\left(c_{j}|M\right)\right)$$

For each condition $c_{j}$, each data-point is also conditionally independent. By the same reasoning:

(Equation 24) $$\text{ln}\left(P\left(c_{j}|M\right)\right)=\overset{n}{\underset{i=1}{\sum }}\text{ln}\left(P\left(d_{i}|M\right)\right)$$

$P\left(d_{i}|M\right)$ is drawn from the p.m.f. generated for the model *M* (i.e. the likelihood of data point $d_{i}$ occurring given model *M*). The model with the greatest log likelihood wins. The results in Table 1 are presented as log likelihood ratios given by:

(Equation 25) $$LR=\text{ln}\left[\frac{P\left(c|M\right)}{P\left(c|\hat{M}\right)}\right]=\text{ln}\left[P\left(c|M\right)\right]-\text{ln}\left[P\left(c|\hat{M}\right)\right]$$

where P(c|M) is the (proportional measure of) likelihood of a model with respect to the data and $P\left(c|\hat{M}\right)$ is the likelihood of the maximally likely model. The maximally likely model gets a score of 0 and scores decrease as candidates become less likely. Akaike Information Criterion (AIC) ratios are also given to compare model fit while penalising parameter counts:

(Equation 26) $$\text{AIC Ratio}=\frac{AIC\left(M\right)}{AIC\left(\hat{M}\right)}$$

with

(Equation 27) AIC(M)=2k−2ln(P(c|M))

where *k* is the number of parameters in the model *M*. The best AIC ratio score is 1 and scores will increase as fit worsens.

As the AIC did not affect the order of our results, we also examined the Bayesian Information Criterion (BIC) as this more heavily penalises large parameter counts. The BIC is defined as:

(Equation 28) BIC(M)=kln(n)−2ln(P(c|M))

with n=564 being the total number of samples against which each model is evaluated (over all conflict conditions), and *k* the number of parameters in the model *M*. These are also reported as ratios in Table 1, given by:

(Equation 29) $$\text{BIC Ratio}=\frac{BIC\left(M\right)}{BIC\left(\hat{M}\right)}$$

with *M* and $\hat{M}$ as above.

### Quantification and statistical analysis

Analysis of circular data was carried out in Oriana 3.21 (Kovach Computing Services, Anglesey, UK) and all presented bearings are shown as μ ± Circular Standard Deviation (SD). All statistical details may be found in the results and discussion. The n-numbers indicate number of individual beetles tested at each experimental condition.

#### Reliability of sun and wind cues

To investigate (i) the beetles’ orientation precision and, (ii) their directional preferences in the presence of a single cue, the ten exit bearings recorded for each beetle were normalised to the azimuthal position of the orientation cue. Beetles whose normalised exits were not significantly different from a uniform distribution (p≤0.05, Rayleigh test) were deemed unable to orient and thus excluded from analysis for tactic behavior. For each experimental group, i.e. elevation of the ersatz sun (except for 90° elevation) or wind speed, Rayleigh tests were conducted on the population of mean bearings. We define menotaxis as mean bearings taken at any angle with respect to the cue (uniform distribution), while a population showing a directional preference towards or away from a directional cue is defined as performing taxis. Orientation precision was then investigated for the experimental groups that performed menotaxis (i.e. beetles that were able to use the sun and wind stimuli as compass cues).

This was done by calculating the mean vector length (*R*) from the normalised bearings of each beetle, including the individuals that were previously excluded. The *R*-value extends from 0 to 1, where a higher value suggests greater precision.

#### Cue conflict between an ersatz sun and wind

To study the effect of cue reliability on the integration and weighting of directional information given by an ersatz sun and wind in conflict, an exclusion criterion was implemented. The criterion stated that, if the six headings were not significantly different from a uniform distribution when the two cues were in their original positions (congruent) (p≥0.1, Rayleigh test, see Figure S2 for justification), then the beetle was eliminated from further analysis. This ensured that the remaining beetles were able to return to their original heading consistently and thus able to orient.

Changes in heading direction were calculated using the angular difference between two consecutive exits (see Figure 2). For changes in heading at the 0° cue conflict, the angular difference was calculated between the first and second exit where the directional information of the two cues remained unchanged (congruent). For changes in heading at a 60° and 120° cue conflict the difference was calculated between an exit where the cues were in congruence and the following exit where the wind cue had been shifted. The population mean change in heading, together with Rayleigh tests (p≥0.05), were used to determine the behavioural response to the azimuthal shift of the wind. This was carried out for all conflict conditions.

To determine individual precision when presented with a conflict between an ersatz sun at a 60° elevation and a 2.5 m/s wind current, the ten bearings recorded at each conflict were tested for uniformity (p≥0.05, Rayleigh test).

## Data Availability

• Behavioral data have been deposited at Zenodo (hosted on GitHub) and are publicly available as of the date of publication. DOIs are listed in the key resources table. • All original code has been deposited at Zenodo (hosted on GitHub) and is publicly available as of the date of publication. DOIs are listed in the key resources table. • Any additional information required to reanalyze the data reported in this paper is available from the lead contact upon request.
