# Supplementary material for: Weighted cue integration for straight-line orientation
Source: iScience. 2022 Sep 30;25(10):105207. doi: 10.1016/j.isci.2022.105207 (PMC9583106; doi:10.1016/j.isci.2022.105207)
Supplement: Document S1. Figures S1–S9 and Table S1 [file mmc1.pdf]

iScience, Volume *25*

Supplemental information

Weighted cue integration

for straight-line orientation

Shahrzad Shaverdian, Elin Dirlik, Robert Mitchell, Claudia Tocco, Barbara Webb, and Marie Dacke

Supplementary material

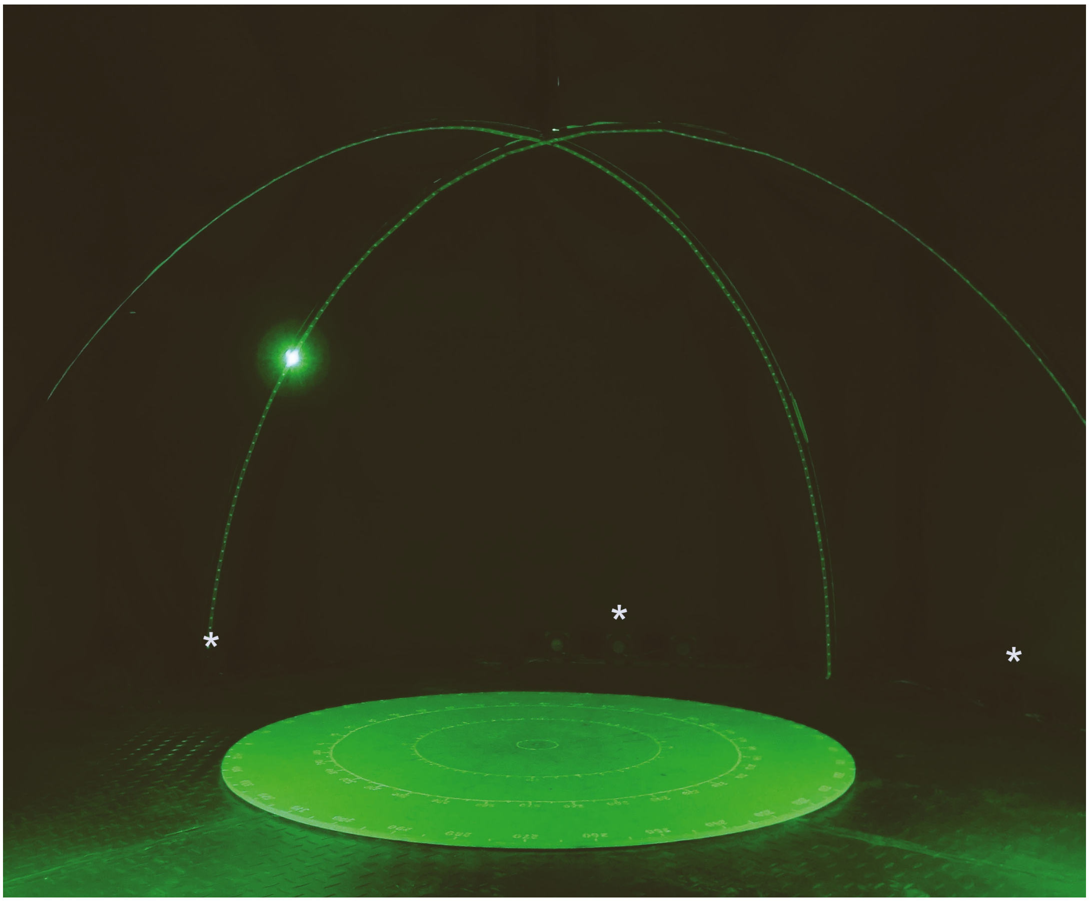

Figure S.1: **The behavioural setup, related to Figures 1 and 2** - The setup used for behavioural experiments, including a circular sand painted arena, two arches lined with green LEDs and three wind generators (each denoted with a white asterisk) positioned at a 0°, 60° and 120° angle relative the green LED used as a sun cue. For detailed information see STAR Methods, Experimental setup.

Table S.1: **The number of beetles tested for tactic behaviour, related to Figure 1** - The number of beetles tested for tactic behaviour at different elevations of an ersatz sun and at different speeds of a wind cue when the ersatz sun was positioned in zenith. Taxis was observed at a 75° solar elevation, as well as at wind speeds 0.5, 0.8, 3.0 and 4.0m/s. The 90° solar elevation has been omitted as there can be no tactic behaviour with respect to a cue in zenith.

Elevation	n	Wind speed	n
5°	18	0.5m/s	10
20°	17	0.8m/s	9
45°	16	1.0m/s	7
60°	17	1.5m/s	14
75°	13	1.9m/s	14
80°	10	2.5m/s	14
82°	15	3.0m/s	13
84°	9	4.0m/s	14
86°	6		
88°	4		

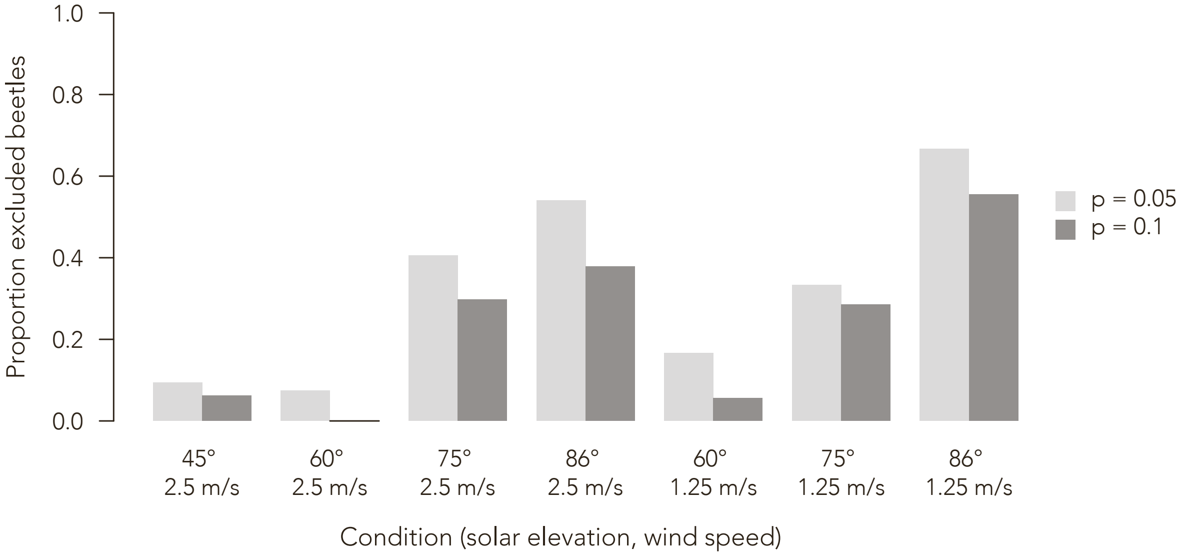

Figure S.2: **The proportion of excluded beetles at cue conflict experiments, related to Figure 2** - To determine the significance threshold for our exclusion criterion, a comparison of the proportion of excluded beetles was carried out for Rayleigh tests using $p = 0.05$ and $p = 0.1$. We found that fewer beetles were excluded when using $p = 0.1$. The behavioural results, i.e. changes in heading, were not different between the two significance levels (p > 0.5, Mardia-Watson-Wheeler tests, $14 \leq n \leq 30$). This held true for all conditions where the Mardia-Watson-Wheeler test was applicable; the number of beetles tested at an 86° solar elevation and a 1.25m/s wind speed was less than 10, which is the minimum number required for the Mardia-Watson-Wheeler test. As the behavioural results were not different between the two significance levels we decided to move forward with the threshold of $p = 0.1$ to maximise the amount of data available for our modelling.

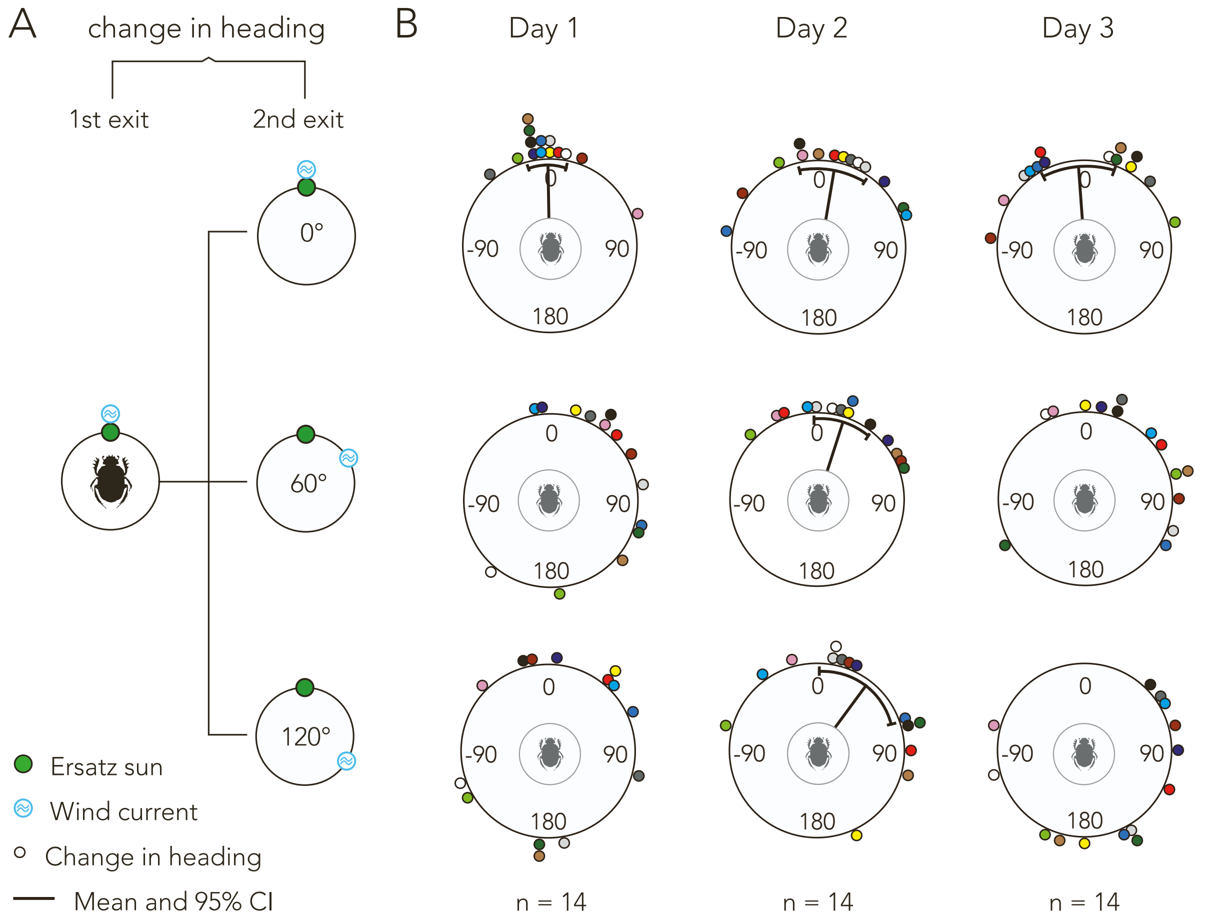

Figure S.3: **Changes in heading of the same individuals across three days, related to Figure 2** - The solar elevation and wind speed were set to 60° and 2.5m/s respectively. A. Schematic procedure of the cue conflict experiment. Change in heading was calculated between two consecutive exits; initial condition (1st exit) to conflict condition (2nd exit). B. Each coloured data point illustrates the change in heading of the same individual across conflicts and days. The line extending from the centre of each circular graph indicates the mean vector and ends in a 95% confidence interval. Note that the data in the third row (120° conflict) is the same as in Figure 2 of this article.

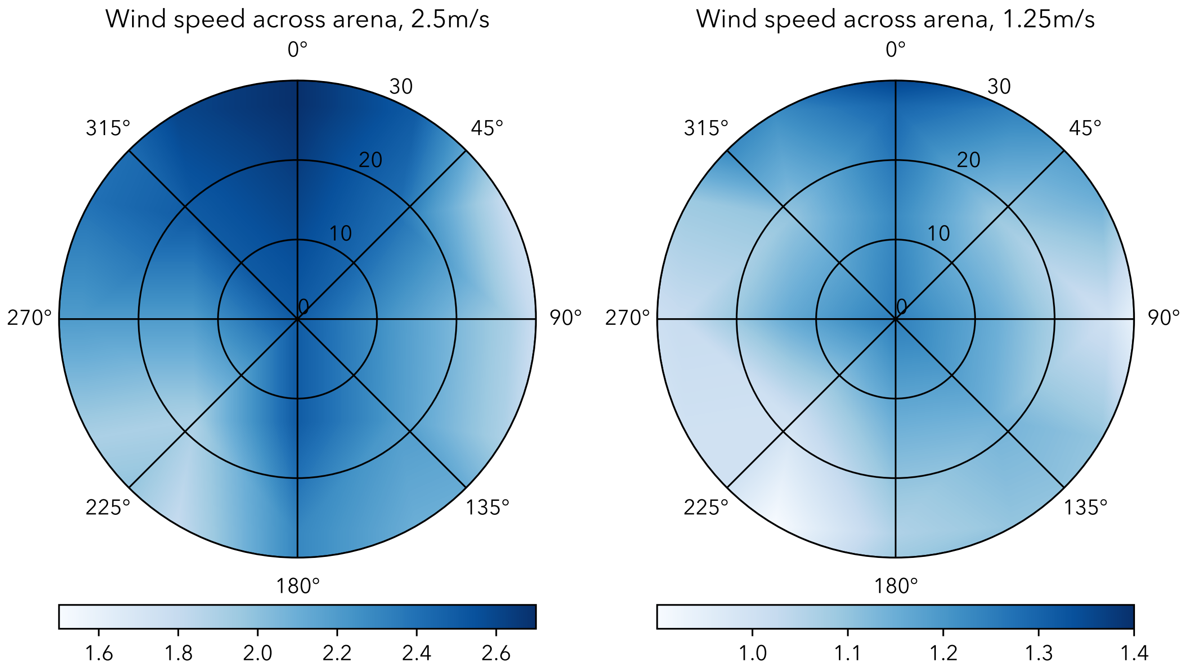

Figure S.4: **Distribution of wind speed, related to Figure 2** - The distribution of wind speeds across our circular arena when the wind speed in the centre is set to 2.5m/s and 1.25m/s. The wind generator is positioned at 0°.

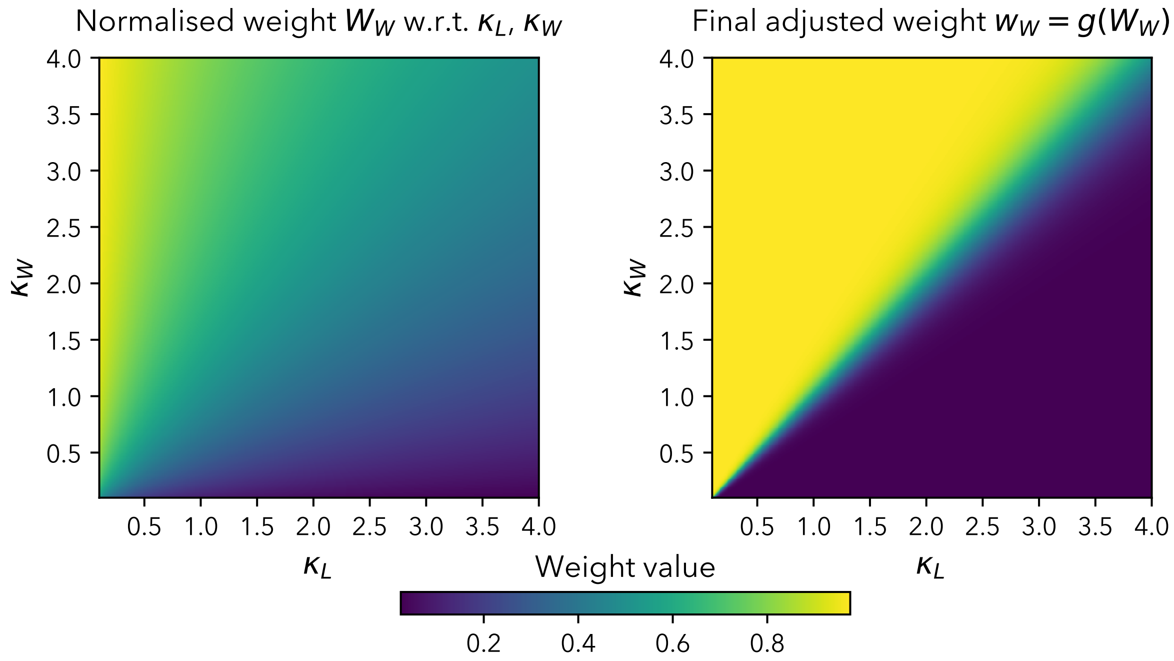

Figure S.5: **The effect of the weight adjustment function** g**, related to STAR Methods (Equation 16)** - The effect of the adjustment function g when applied to the normalised weight W_{Wind}. After adjustment, the cue with greater weight will dominate the integration. Weights are defined w.r.t. kappas for the cue distributions, see STAR Methods, Integration models.

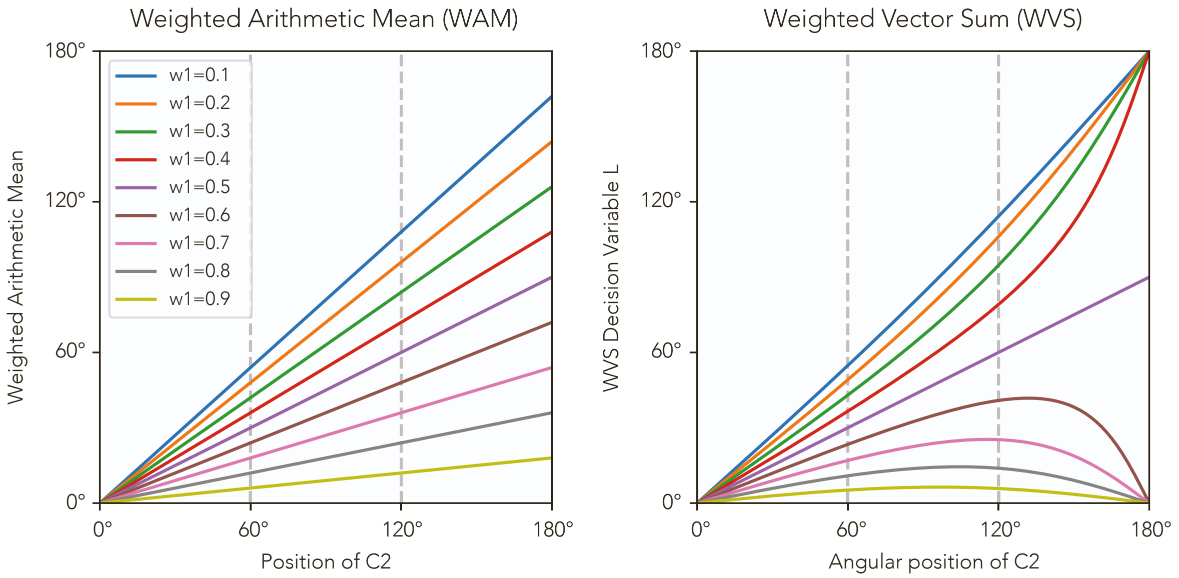

Figure S.6: **Illustration of the difference between WAM and WVS outputs for different conflicts and weights, related to STAR Methods (Equations 10 and 13)** - Adapted from (Murray and Morgenstern, 2010). The contrasting outputs of the linear weighted arithmetic mean and the circular weighted vector sum. The difference between the two strategies becomes more striking at large conflicts, especially when observing noisy populations (see Figure 3). Experimental conflicts of 60° and 120° shown with dashed lines.

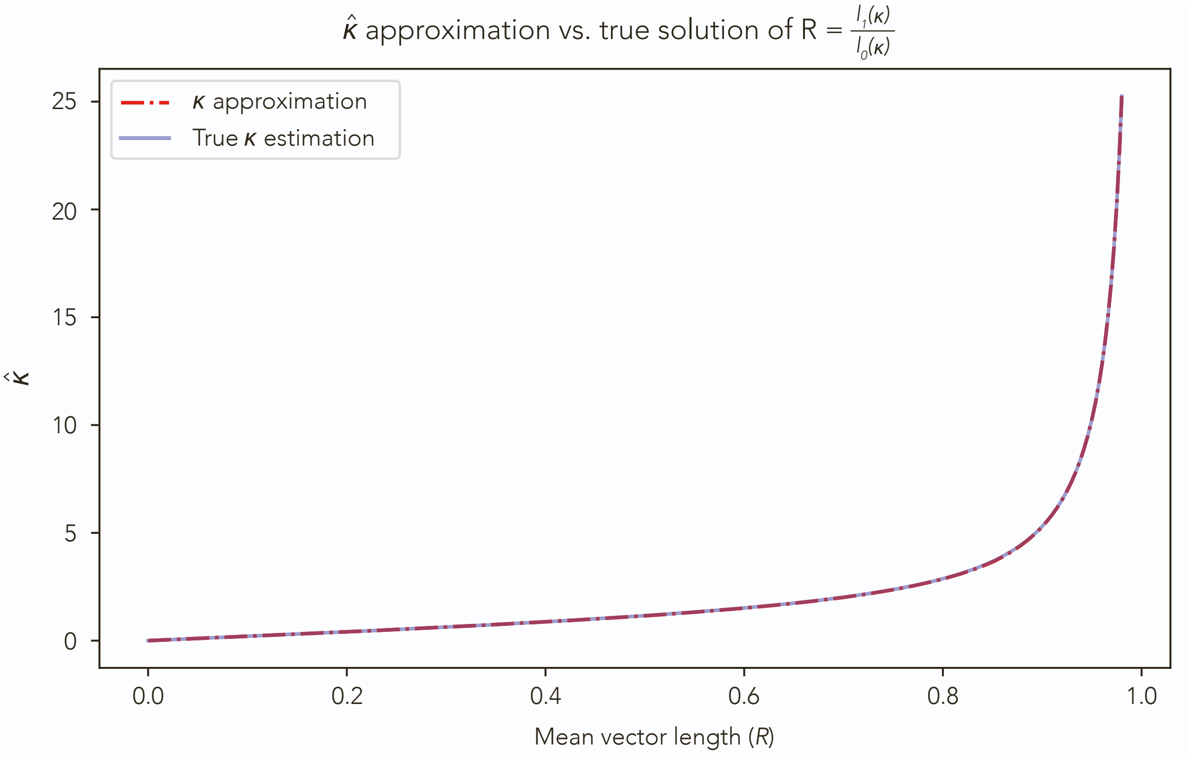

Figure S.7: **The κ approximation from Mardia and Jupp (2009) (Red, dot-dash) compared to the true MLE for κ given by solutions to $R = \frac{I_1(\kappa)}{I_0(\kappa)}$ (Blue, solid), related to STAR Methods (Equation 4)** - We opted for the approximation as it was slightly faster to compute and (as can be seen) sufficiently accurate. The approximation is due to Mardia and Jupp (2009); note that for large R we specifically use Equation 5.3.8 from (Mardia and Jupp, 2009) as it provides a better approximation than their final expression in Equation 5.3.9 (tested in the same manner, by comparing to the true solutions).

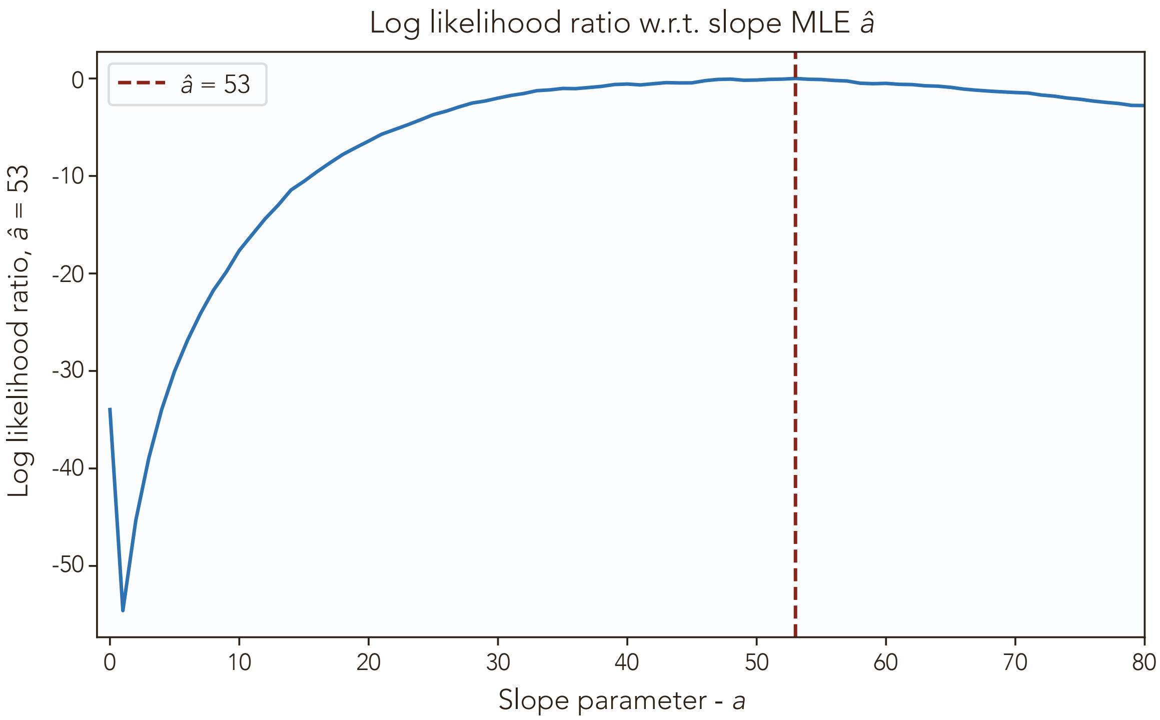

Figure S.8: **NVS parameter analysis; selection of parameter *a* in the weight adjustment function, related to STAR Methods (Equation 16)** - In order to choose a slope parameter a we performed the same likelihood analysis on NVS across a range of parameters. For convenience we defined $a = 0$ such that $g(x; 0) = x$; thus, varying a gives an effective spectrum from 'optimal' weights to winner-take-all. As can be seen, our MLE lies somewhere in the middle of this spectrum, indicating that while one cue will tend to dominate, both are always considered. This analysis indicated the maximally likely slope $\hat{a} = 53$. The flat nature of the peak and the randomness inherent in the population generation process means that $\hat{a}$ would be likely to change if the analysis were run again, however it would remain in the same general area.

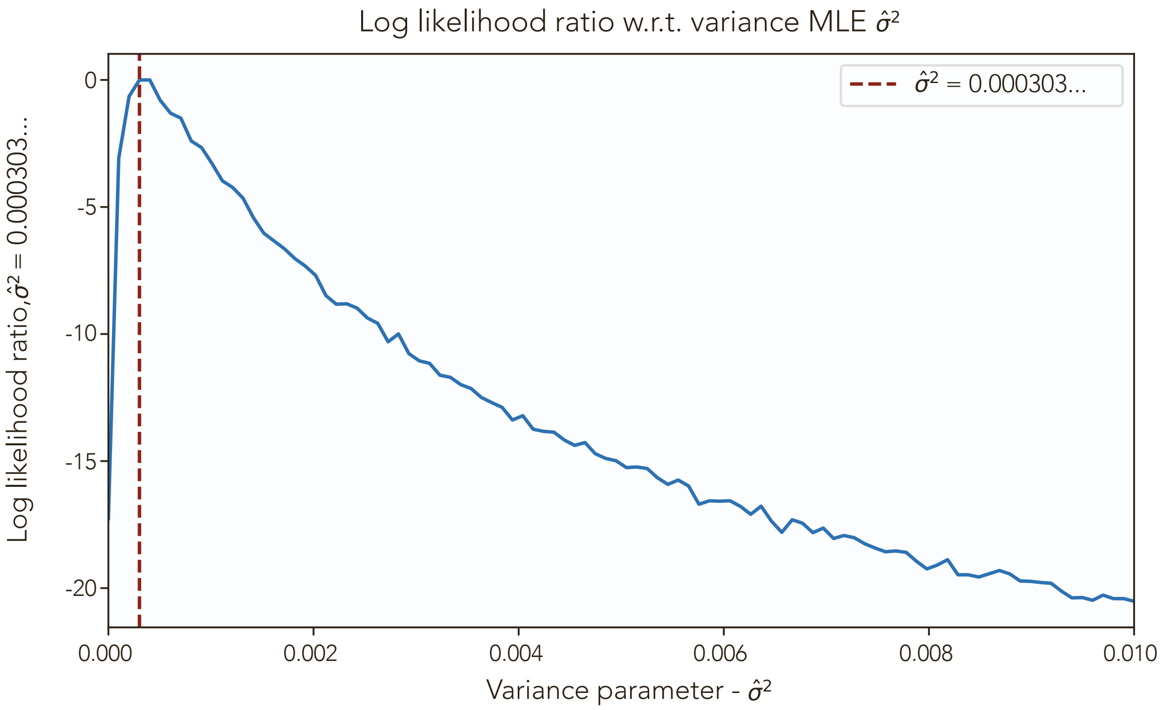

Figure S.9: **BVS parameter analysis; selection of σ^2 for Gaussian bias distributions, related to STAR Methods (Equations 17 and 18)** - In order to choose a representative bias variance σ^2, we performed a likelihood analysis on BVS across a range of parameters. σ^2 is the variance of the Gaussian distribution from which the biases are drawn. This analysis indicated a maximally likely variance $\hat{\sigma}^2 = 0.000303$. Again this is subject to change if the full analysis were to be run again, however the narrow peak indicates this value would be more stable than the slope parameter.
